# Genetic susceptibility of common polymorphisms in *NIN* and *SIGLEC5* to chronic periodontitis

**DOI:** 10.1038/s41598-019-38632-5

**Published:** 2019-02-14

**Authors:** Hua Tong, Zhuliang Wei, Jing Yin, Bo Zhang, Tianxiao Zhang, Chunni Deng, Yali Huang, Nan Zhang

**Affiliations:** 1 Department of Stomatology, the First Affiliated Hospital, School of Medicine, Xi’an Jiaotong University, Xi’an, China; 2 Department of Anesthesiology, the First Affiliated Hospital, School of Medicine, Xi’an Jiaotong University, Xi’an, China; 3 Department of Stomatology, Jinan Stomatological Hospital, Jinan, China; 4 Department of Biomedical Engineering, School of Life Science and Technology, Xi’an Jiaotong University, Xi’an, China; 5 Department of Epidemiology and Biostatistics, Health Science Center, Xi’an Jiaotong University, Xi’an, China

## Abstract

Chronic periodontitis (CP) is a common oral disease characterized by the slow progression of alveolar attachment loss and bone destruction. Genetic components have been reported to play an important role in the onset and development of CP. In the present study, we aimed to replicate the association signals of *NIN* and *SIGLEC5* identified in previous genome-wide association studies (GWASs) of samples from Chinese Han individuals. Association signals between clinical severity indicators of CP and relevant single nucleotide polymorphisms (SNPs) were also examined. A total of 3,160 study subjects, including 1,076 CP patients and 2,084 healthy controls, were recruited. A total of 32 SNPs, including 22 from *NIN* and 10 from *SIGLEC5*, were selected for genotyping. SNPs rs12883458 (OR = 1.45, *P* = 1.22 × 10^−5^, *NIN*) and rs4284742 (OR = 0.75, *P* = 1.69 × 10^−5^, *SIGLEC5*) were significantly associated with CP disease status. rs4284742 was significantly associated with all 3 clinical severity indicators, including bleeding on probing (BOP), probing depth (PD) and clinical attachment loss (CAL). According to evidence from bioinformatics analyses, both significant SNPs, rs12883458 and rs4284742, are likely surrogates of underlying variants with true effects. In summary, our findings provide direct evidence for the association of *NIN* and *SIGLEC5* with CP susceptibility.

## Introduction

Chronic periodontitis (CP) is a common oral disease characterized by the slow progression of alveolar attachment loss and bone destruction^1,2^. The prevalence of CP varies among different populations, ranging from 40% to 80%; this variance is likely due to differences in diagnostic criteria and methodology^3–6^. CP has severe impacts on quality of life and is considered one of the major causes of tooth loss in adults^7,8^. In addition, CP is also linked to other conditions, including Alzheimer’s disease^9^, head and neck squamous cell carcinoma^10^, coronary heart disease^11^ and pregnancy outcomes^12^.

Several risk factors, including age, race, obesity and smoking status, have been shown to contribute to the risk of CP^6,13–15^. Genetic components have been reported to play an important role in the onset and development of CP^16,17^. Early family-based studies have estimated that approximately 50% of phenotypic variations of CP could be explained by genetic factors^17^. A few genome-wide association studies (GWASs) have been published, and several susceptible loci, including *SIGLEC5*^18^, *DEFA1A3*^18^, *NIN*^19–22^, *NPY*^19^, *WNT5A*^19^, *NCR2*^19^, *EMR1*^19^, *ABHD12B*^20^, *WHAMM*^20^ and *AP3B2*^20^, have been found to be associated with CP status and periodontal health. Although these previous GWASs have provided novel insights into the potential genetic etiology of CP and identified several candidate susceptible genes, further research is still needed due to the challenges inherent to GWASs, including a low portion of heritability explained by identified loci and non-consideration of biological and pathological mechanisms.

Ninein is a centrosomal microtubule organization and anchoring protein encoded by the *NIN* gene. Cytotoxic T lymphocytes, which are strongly related to the pathogenesis of CP, are functionally associated with the centrosome. In this sense, a potential link in biological mechanisms can be drawn between *NIN* and CP. At least two previous GWASs have reported genetic connections between *NIN* and CP^19,20^. Sialic acid-binding immunoglobulin-like lectins (SIGLECs) are a group of immunoglobulin families that mediate protein-carbohydrate interactions. Siglec 5 protein has been identified to be present mainly in neutrophils and plays a regulatory role in the activation of myeloid cells in the prevention of inappropriate reactivity against self tissues^23,24^. A recently published GWAS linked *SIGLEC5* to aggressive periodontitis^18^. Because the underlying molecular mechanisms of periodontitis are still largely unclear, the effects of the *NIN* and *SIGLEC5* genes on periodontitis have not been clarified, although significant associations with periodontitis were identified in the European population. Because the roles of the *NIN* and *SIGLEC5* genes in periodontitis susceptibility in Han Chinese individuals have not previously been evaluated, we performed a case-control study to assess the relationship of the *NIN* and *SIGLEC5* genes with periodontitis susceptibility in Han Chinese individuals to improve our current understanding of the roles of these genes in periodontitis predisposition. In the present study, we aimed to explore the relationship of the association signals of the *NIN* and *SIGLEC5* genes identified in a previous GWAS with CP in a Han Chinese population, and association signals between clinical severity indicators of CP and relevant single nucleotide polymorphisms (SNPs) were also examined.

## Methods

### Study subjects

The subjects consisted of 1,076 CP patients and 2,084 healthy controls ranging from 36 to 64 years of age. All subjects were unrelated Han Chinese individuals from Shaanxi Province and were recruited from the inpatient and outpatient clinical services at the First Affiliated Hospital of Xi’an Jiaotong University between May 2014 and November 2016. The diagnosis of CP was made based on the criteria established in 1999 at the World Workshop for a Classification of Periodontal Diseases and Conditions. The healthy controls were enrolled according to the following criteria: (i) probing depth ≤3 mm; (ii) absence of any clinical signs of gingival inflammation; and (iii) no history of periodontal disease. Individuals who met the following criteria were excluded from the present study: (i) current and former smokers; (ii) pregnant or lactating women; (iii) use of antibiotics or anti-inflammatory medications in the past six months; (iv) severe medical disorders that could influence periodontal pathogenesis and/or treatment outcomes; (v) modifiers of periodontal disease, such as rheumatoid arthritis, diabetes and osteoporosis; and (vi) periodontal therapy performed within the previous six months. The clinical parameters of each subject were recorded. Bleeding on probing (BOP), probing depth (PD) and clinical attachment loss (CAL) were measured at six sites per tooth using a manual probe (UNC-15, Hu-Friedy Manufacturing Company, Inc., Chicago, IL, USA). All four examiners were dentists and performed examinations using a manual probe (UNC-15, Hu-Friedy Manufacturing Company, Inc., Chicago, IL, USA). All examiners were calibrated on a set of 40 patients probed twice before examinations were conducted in this study. We used a kappa statistic to measure agreement between different examiners, and the kappa index and intraclass correlation coefficient for PD ranged from 0.80 to 0.93 and 0.76 to 0.89, respectively, for intraexaminer calibration. The interexaminer kappa index and intraclass correlation coefficient ranged from 0.83 to 0.92 and 0.79 to 0.87, respectively, for PD (Supplemental Table S1). These results ensured the reliability of clinical examinations. Characteristic information of these study subjects is summarized in Table 1. No significant difference between cases and controls was identified for age (*P* = 0.73) and gender (*P* = 0.93). Informed consent was obtained from all subjects. The study protocol conformed to the ethical guidelines of the Declaration of Helsinki (version 2002) and was approved by the Ethical Committee of Xi’an Jiaotong University.

**Table 1:** Demographic and clinical characteristics of the subjects.

Variables	Study Subjects (N = 3,160)		Statistics	*P*-value
	Patients (N = 1,076)	Controls (N = 2,084)		
Age (years), mean ± SD	43.06 ± 7.17	43.16 ± 7.02	*T* = −0.35	0.73
**Gender** (**%**)				
*Male*	745 (69)	1,440 (69)		
*Female*	331 (31)	644 (31)	χ^2^ = 0.0016	0.97
No. of lost teeth, mean ± SD	2.10 ± 1.07	0.30 ± 0.46	—	—
BOP (%)	80.07 ± 4.70	13.44 ± 3.27	—	—
PD (mm), mean ± SD	6.27 ± 0.86	1.82 ± 0.24	—	—
CAL(mm), mean ± SD	5.30 ± 0.82	0.51 ± 0.18	—	—
**Clinical severity** (**%**)				
*Moderate (3* ≤ *CAL* < *5)*	373 (35)	—	—	—
*Severe (CAL* ≥ *5)*	703 (65)	—	—	—

SD: standard deviation; BOP: bleeding on probing; PD: probing depth; CAL: clinical attachment loss.

### *SNP* selection and genotyping

Tag SNPs with a minor allele frequency (MAF) ≥ 0.05 located within the gene regions of *NIN* and *SIGLEC5* were selected for genotyping. The pairwise R^2^ threshold used for tagging was 0.6. A total of 32 SNPs, including 22 from *NIN* and 10 from *SIGLEC5*, were selected for genotyping. Basic information for these 32 SNPs, including the MAF and Hardy-Weinberg equilibrium (HWE) test results, is summarized in Supplemental Table S2. Genomic DNA was isolated from peripheral blood using a Tiangen DNA extraction kit (Tiangen Biotech Co. Ltd, Beijing, China) according to the manufacturer’s protocol. SNP genotyping was performed using a Sequenom MassARRAY platform with iPLEX GOLD chemistry (Sequenom, San Diego, CA, USA) based on the manufacturer’s protocol. The results were processed using Sequenom Typer 4.0 software, and genotype data were generated from the samples. Genotyping was conducted by laboratory personnel blinded to the case-control status, and the genotyping results, data entry and statistical analyses were independently reviewed by two authors. We randomly reperformed the analysis on 5% of the sample, which resulted in a concordance of 100%.

### Statistical analyses

Single marker-based associations were examined by performing χ^2^ tests for each SNP. We performed a one degree of freedom test (allelic test) for single marker-based association analyses. An additive mode of inheritance was assumed, and the effect of the minor allele was tested for each marker. Linkage disequilibrium (LD) blocks were constructed, and haplotype-based association analyses were performed. Stratification analyses were then performed according to the clinical severity of CP. CP patients with CAL ≥ 5 mm were defined as severe CP patients, while those with 3 mm ≤ CAL < 5 mm were defined as moderate CP patients. In addition, we also performed association analyses between significant SNPs and several clinical severity indicators of CP, including BOP, PD and CAL, using our CP samples. Linear regression models were fitted for this analysis. BOP, PD and CAL were used as phenotypes, and genotypes were used as variables. An additive mode of inheritance was assumed, and genotypes of each marker were coded as 0, 1 and 2 when 0, 1 and 2 minor alleles were present. Gene-gene interactions were also investigated by case-only analyses^25^. LD block construction and haplotype analyses were conducted using Haploview^26^. LD blocks were constructed by an algorithm proposed by Gabriel *et al*.^27^. Haplotype-based association analyses were then performed for each LD block. Haplotypes were estimated by an expectation maximization algorithm. Counts for case-control association tests were obtained by summing the fractional likelihoods of each individual for each haplotype. Single marker-based association, stratification, clinical severity association and gene-gene interaction analyses were performed by Plink^28^. Bonferroni corrections were applied for multiple comparisons. The *P* value threshold used for single marker-based analyses was 0.05/32≈0.0016.

### Bioinformatics analyses

We examined the potential functional consequences of significant SNPs using data from the GTEx database (https://www.gtexportal.org/home/)^29^. Expression quantitative loci (eQTL) data of significant SNPs on *NIN* and *SIGLEC5* from 47 human tissues were extracted.

## Results

### Genetic associations for CP status

Significant signals were identified from both candidate loci (Table 2, Supplemental Table S3). SNPs rs12883458 (OR = 1.45 *P* = 1.22 × 10^−5^, *NIN*) and rs4284742 (OR = 0.75, *P* = 1.69 × 10^−5^, *SIGLEC5*) were significantly associated with CP disease status. A Q-Q plot for the results of genetic association analyses is shown in Supplemental Fig. S1. Four and one LD blocks were constructed within the gene regions of *NIN* and *SIGLEC5*, respectively (Supplemental Fig. S3–4). The results of haplotype analyses replicated the significant hit from *SIGLEC5*. A two-SNP haplotype, rs2278831-rs4284742, was significantly associated with CP status (*P* = 1.29 × 10^−9^, Supplemental Table S4).

**Table 2:** Significant SNPs identified in single marker-based association analyses.

SNP	Chr	A1	Gene	Genotypes	Patients	Controls	χ^2^	*P*	OR
rs12883458	14	C	*NIN*	CC (N = 42)	21	21	19.13	1.22 × 10^−5^	1.45
				CT (N = 541)	220	321			
				TT (N = 2,577)	835	1,742			
rs4284742	19	A	*SIGLEC5*	AA (N = 141)	33	108	18.51	1.69 × 10^−5^	0.75
				AG (N = 1,002)	306	696			
				GG (N = 2,017)	737	1,280			

Chr: chromosome; A1: tested allele.

### Differential effects of multiple SNPs from *SIGLEC5* in moderate and severe *CP* patients

Stratification analyses were performed for moderate and severe CP patients. Significant results are summarized in Table 3. SNP rs12883458 on *NIN* had similar effects in both the moderate and severe CP groups. Interestingly, 6 SNPs on *SIGLEC5*, including the significant hit rs4284742, showed differential patterns of their effects in the moderate and severe CP groups. The minor alleles of these SNPs increased the risk of CP in the moderate CP group, while the same alleles were protective in the severe CP group. In addition to the stratification analyses, we also conducted case-only analyses for the moderate and severe CP groups to estimate the differential effects of the SNPs of *SIGLEC5*. Severe CP was coded as 2, and moderate CP was coded as 1. The results are summarized in Supplemental Table S5. These results were very similar to the results of the stratification analyses presented in Table 3.

**Table 3:** Significant SNPs identified in single marker-based association analyses stratified by clinical severity of chronic periodontitis.

Gene	SNP	Chr	A1	Moderate (N = 2,457)			Severe (N = 2,787)		
				χ^2^	*P*	OR	χ^2^	*P*	OR
*SIGLEC5*	rs2278831	19	G	32.99	9.27 × 10^−9^	1.74	25.07	5.52 × 10^−7^	0.61
*SIGLEC5*	rs556418978	19	T	23.18	1.47 × 10^−6^	1.79	7.28	0.0070	0.71
*SIGLEC5*	rs4801882	19	G	20.88	4.89 × 10^−6^	1.44	13.11	0.0003	0.80
*SIGLEC5*	rs73050865	19	G	15.66	7.57 × 10^−5^	1.75	3.62	0.0573	0.75
*SIGLEC5*	*rs4284742	19	A	14.71	0.0001	1.41	73.58	9.68 × 10^−18^	0.46
*NIN*	*rs12883458	14	C	10.61	0.0011	1.49	13.35	0.0003	1.43
*SIGLEC5*	rs4802831	19	C	4.84	0.0278	1.37	7.31	0.0068	0.68

Chr: chromosome; A1: tested allele.

*Significant SNPs identified in single marker-based association analyses.

### Significant associations between susceptible SNPs and clinical severity of *CP*

**Figure 1:**
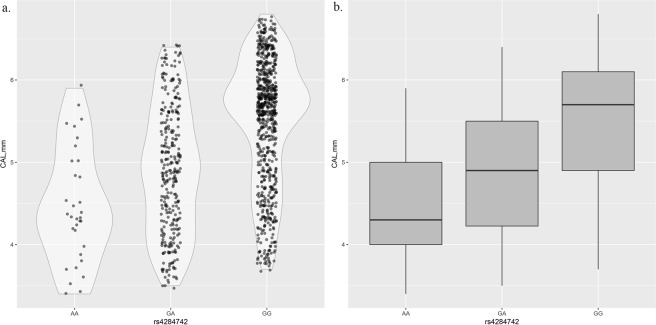
Relationship between genotypes of rs4284742 and clinical attachment loss. (**a**) Violin plot for genotypes of rs4284742 and clinical attachment loss. (**b**) Box plot for genotypes of rs4284742 and clinical attachment loss.

We performed association analyses between the significant SNPs, rs12883458 and rs4284742, and the clinical severity of CP, including BOP, PD and CAL, using CP samples. The results of this association analysis are summarized in Table 4. rs4284742 was significantly associated with all 3 clinical severity indicators. No significant signals were obtained for rs12883458. The association patterns between rs4284742 and CAL are shown in Fig. 1. The average CAL significantly increased when allele *G* (major allele) of rs4284742 was present (*P* = 5.92 × 10^−37^).

**Table 4:** Association between significant SNPs and clinical severity measurements of chronic periodontitis in 1,076 cases.

SNP	Chr	A1	Gene	BOP				PD			CAL	
				*β*	STAT	*P*	*β*	STAT	*P*	*β*	STAT	*P*
rs12883458	14	C	*NIN*	0.45	1.47	0.1423	−0.01	−0.11	0.9154	0.01	0.19	0.8486
rs4284742	19	A	*SIGLEC5*	−2.35	−9.13	3.43 × 10^−19^	−0.38	−8.04	2.34 × 10^−15^	−0.57	−13.20	5.92 × 10^−37^

Chr: chromosome; BOP: bleeding on probing; PD: probing depth; CAL: clinical attachment loss.

### Gene-gene interactions and bioinformatics analyses

No significant SNP pairs were obtained in our gene-gene interaction analyses (Supplemental Table S6). Although several SNP pairs showed nominal significance, they were not significant after corrections for multiple comparisons. In addition, no significant differences were identified for both rs12883458 and rs4284742 in the gene expression of *NIN* and *SIGLEC5* in multiple human tissues (Supplemental Table S7–8).

## Discussion

In the present study, we identified a significant association between two SNPs, rs12883458 and rs4284742, and the disease status of CP in a Chinese population. Our findings successfully replicated loci reported by several previous GWASs^18–20^. To the best of our knowledge, this study is the first genetic association study of CP focusing on the *NIN* and *SIGLEC5* genes based on samples from individuals with Chinese Han ancestry. Both the direction and size of the significant SNPs were similar to those of previous studies conducted on European populations. Both significant SNPs are located within the intronic region and therefore might not be related to the structural alterations of the protein products. Further bioinformatics analyses based on eQTL data extracted from the GTEx database showed that neither SNP was significantly associated with gene expression in multiple human tissues. Thus, the likelihood that the two SNPs were eQTL is very low. Combining all of these findings, we could conclude that both significant SNPs, rs12883458 and rs4284742, might be surrogates of other underlying variants with true effects. As this work is a candidate gene-based genetic association study using common polymorphisms, clearly identifying the variants with true effects based on data from this study is difficult. Nevertheless, the results from stratification analyses could provide some information from this work. In addition to rs4284742, we identified 5 other SNPs of *SIGLEC5* that were significantly associated with CP status in both the moderate and severe CP groups. Interestingly, the most significant SNP in the moderate CP group was not rs4284742 but rs2278831, which is a nonsynonymous variant of *SIGLEC5* (Phe322 Ser). This SNP altered the amino acid sequence of the protein product encoded by *SIGLEC5* and might therefore be a potential candidate for a true effective variant. In recent decades, with the fast developments of high-throughput technologies, candidate gene association studies have successfully identified many susceptibility locus for complex diseases^30–40^. Nevertheless, the data of our single study were not sufficient to confirm this hypothesis. Further studies, especially studies based on sequencing technology, are still needed in the future to systematically unravel the genetic architecture of CP.

In the stratification analyses, we identified SNP rs4284742, and the other 5 SNPs of *SIGLEC5* had differential patterns of effects in moderate and severe CP patients. The minor alleles of these SNPs were significantly related to elevated CP risk in moderate CP cases, while these same minor alleles had protective effects in the severe CP group (5 of 6 were significant). This observation has never been reported in previous studies, although stratification analyses by severity of CP have often been conducted^18–20^. One possible explanation for this observation is that *SIGLEC5* might play different roles in the disease pathology in moderate and severe CP. A hypothesis is that the associated *SIGLEC5* variants might affect the progression of CP from moderate to severe, rather than the risk of disease. Another explanation is that the estimated LD structures of these reported SNPs and the underlying variants with true effects were not similar between the moderate and severe CP groups in the study. In the stratification analyses, each group had fewer samples than the full sample set. Therefore, the estimation of LD structures might be significantly altered. In this sense, the patterns of association signals would be altered in turn. Future replication studies are needed to validate both assumptions. If our findings of this differential pattern cannot be replicated by other studies, then the differential pattern of association signals is likely explained by the decreased sample size in the stratification analyses. However, if this differential pattern in the effect direction of rs4284742 could be replicated by several follow-up studies, then this SNP could play a role in the underlying pathological mechanisms of CP.

Associations between both significant SNPs and clinical severity indicators of CP identified a significant hit for rs4284742. The major allele of rs4284742, allele *G*, was significantly associated with elevated levels of BOP, PD and CAL. Each copy of the *G* allele could increase the CAL level by approximately 0.6 on average. This significant signal was not identified for rs12883458 of *NIN*. Our findings indicated that rs4284742 not only contributed to the risk of CP but also was related to an elevated level of CP severity.

This study had several limitations. First, in this study, we only genotyped SNPs located within the gene regions of *NIN* and *SIGLEC5*. However, regions several kb up/downstream of these genes have been proven to be very important regulatory regions. Omitting these regions would reduce the genetic information coverage and may miss some potential significant hits. Second, we did not apply any procedures to control population stratification, which is a major confounder in genetic association studies. According to the Q-Q plot of the results of the genetic association analyses, no systematic inflation of *P* value significance could be observed. Nevertheless, drawing conclusions regarding population stratification from a Q-Q plot made from genotyping variants of two genes is insufficient.

## Conclusion

In the present study, we found SNPs of *NIN* and *SIGLEC5* to be significantly associated with CP. SNPs in *SIGLEC5* were also found to be significantly related to the clinical severity of CP. According to evidence from bioinformatics analyses, both significant SNPs, rs12883458 and rs4284742, are likely surrogates of underlying variants with true effects. Studies based on sequencing technology are still needed in the future to investigate the true effective markers.

## Supplementary information

Supplemental Materials
