# Supplementary material for: Genetic susceptibility of common polymorphisms in NIN and SIGLEC5 to chronic periodontitis
Source: Sci Rep. 2019 Feb 14;9:2088. doi: 10.1038/s41598-019-38632-5 (PMC6376118; doi:10.1038/s41598-019-38632-5)
Supplement: Supplementary file 1 — Supplemental Materials [file 41598_2019_38632_MOESM1_ESM.docx]

***Title:*** Genetic susceptibility of common polymorphisms in *NIN* and *SIGLEC5 to* chronic periodontitis

***Author names and affiliations***: Hua Tong ^a, b *^, Zhuliang Wei ^c *^, Jing Yin ^c *^, Bo Zhang ^d^, Tianxiao Zhang ^e^, Chunni Deng ^a^, Yali Huang ^a^ and Nan Zhang ^a^

^a^ Department of Stomatology, the First Affiliated Hospital, School of Medicine, Xi’an Jiaotong University, Xi’an, China

^b^ Department of Anesthesiology, the First Affiliated Hospital, School of Medicine, Xi’an Jiaotong University, Xi’an, China

^c^ Department of Stomatology, Jinan Stomatological Hospital, Jinan, China;

^d^ Department of Biomedical Engineering, School of Life Science and Technology, Xi'an Jiaotong University, Xi’an, China;

^e^ Department of Epidemiology and Biostatistics, Health Science Center, Xi’an Jiaotong University, Xi’an, China.

* These authors contributed equally to this work.

***Corresponding Author***:

Nan Zhang, Ph.D., M.D., Department of Stomatology, the First Affiliated Hospital, School of Medicine, Xi’an Jiaotong University, 277 Yanta West Road, Xi’an, China, 710061.

Tel: +86-29-85324156, Fax: +86-29-85324141, E-mail: [nanzhangnz@126.com](mailto:nanzhangnz@126.com)

Supplemental Table S1 Results of intra- and inter-examiner calibration for pocket depth

| Trainee | Intra-examiner calibration | | Inter-examiner calibration | | | | | |
| --- | --- | --- | --- | --- | --- | --- | --- | --- |
|  | K-test | ICC | K-test | ICC | K-test | ICC | K-test | ICC |
| 1 | 0.87 | 0.84 | 2: 0.83 | 2: 0.79 | 3: 0.90 | 3: 0.83 | 4: 0.85 | 4: 0.82 |
| 2 | 0.80 | 0.76 | 3: 0.91 | 3: 0.85 | 4: 0.86 | 4: 0.81 |  |  |
| 3 | 0.84 | 0.79 | 4: 0.92 | 4: 0.87 |  |  |  |  |
| 4 | 0.93 | 0.89 |  |  |  |  |  |  |

K-test = Kappa test; ICC = Intra-class Coefficient Correlation

Supplemental Table S2. Basic information for 32 selected SNPs.

| CHR | POS | SNP | Alleles | FUNC | Loci | MAF | HWE |
| --- | --- | --- | --- | --- | --- | --- | --- |
| 14 | 50725016 | rs1599969 | C/T | intron | *NIN* | 0.34 | 0.81 |
| 14 | 50752053 | rs7494766 | A/G | intron | *NIN* | 0.47 | 0.97 |
| 14 | 50752631 | rs61755995 | A/G | missense | *NIN* | 0.16 | 0.57 |
| 14 | 50757699 | rs2236316 | C/G | missense | *NIN* | 0.14 | 0.59 |
| 14 | 50758414 | rs2073349 | G/T | coding-synon | *NIN* | 0.48 | 0.51 |
| 14 | 50764949 | rs77708675 | A/C | intron | *NIN* | 0.15 | 0.49 |
| 14 | 50789519 | rs72683629 | A/C | intron | *NIN* | 0.08 | 1.00 |
| 14 | 50795433 | rs17122909 | C/T | intron | *NIN* | 0.18 | 1.00 |
| 14 | 50801132 | rs12886246 | C/T | intron | *NIN* | 0.48 | 0.97 |
| 14 | 50802518 | rs34281449 | G/T | intron | *NIN* | 0.29 | 0.67 |
| 14 | 50802558 | rs72683633 | A/G | intron | *NIN* | 0.10 | 0.91 |
| 14 | 50811717 | rs113337019 | C/G/T | intron | *NIN* | 0.11 | 0.45 |
| 14 | 50812661 | rs12883458 | C/T | intron | *NIN* | 0.10 | 0.17 |
| 14 | 50813954 | rs7153720 | C/T | intron | *NIN* | 0.28 | 0.59 |
| 14 | 50815094 | rs77367944 | A/G | intron | *NIN* | 0.26 | 0.82 |
| 14 | 50816430 | rs7149295 | C/T | intron | *NIN* | 0.36 | 0.67 |
| 14 | 50819411 | rs10132475 | A/G | intron | *NIN* | 0.15 | 0.86 |
| 14 | 50822684 | rs1951476 | C/T | intron | *NIN* | 0.13 | 0.45 |
| 14 | 50823156 | rs1547077 | G/T | intron | *NIN* | 0.16 | 0.74 |
| 14 | 50826026 | rs3759525 | A/G | intron | *NIN* | 0.09 | 1.00 |
| 14 | 50826321 | rs8018156 | A/G | intron | *NIN* | 0.10 | 0.19 |
| 14 | 50828782 | rs8010709 | G/T | intron | *NIN* | 0.39 | 0.46 |
| 19 | 51612691 | rs73050865 | C/G | intron | *SIGLEC5* | 0.06 | 0.40 |
| 19 | 51619941 | rs73050880 | A/G | intron | *SIGLEC5* | 0.06 | 0.20 |
| 19 | 51623800 | rs4801882 | A/G | intron | *SIGLEC5* | 0.48 | 0.83 |
| 19 | 51623881 | rs4802831 | C/T | intron | *SIGLEC5* | 0.06 | 0.48 |
| 19 | 51627084 | rs8105105 | C/T | intron | *SIGLEC5* | 0.23 | 0.58 |
| 19 | 51627235 | rs17740650 | A/G | coding-synon | *SIGLEC5* | 0.38 | 0.71 |
| 19 | 51627866 | rs2278831 | A/G | missense | *SIGLEC5* | 0.15 | 0.66 |
| 19 | 51628480 | rs4284742 | A/G | intron | *SIGLEC5* | 0.20 | 0.31 |
| 19 | 51628608 | rs556418978 | C/T | intron | *SIGLEC5* | 0.08 | 0.28 |
| 19 | 51629807 | rs2278833 | G/T | intron | *SIGLEC5* | 0.13 | 0.28 |

HWE: *P* values for tests of Hardy-Weinberg equilibrium in control samples only.

Supplemental Table S3. Full results for single marker based association study.

| CHR | SNP | POS | A1 | F_A | F_U | χ^2^ | *P* | OR | Loci |
| --- | --- | --- | --- | --- | --- | --- | --- | --- | --- |
| 14 | rs1599969 | 50725016 | T | 0.33 | 0.34 | 0.07 | 0.80 | 0.99 | *NIN* |
| 14 | rs7494766 | 50752053 | G | 0.47 | 0.47 | 0.13 | 0.72 | 0.98 | *NIN* |
| 14 | rs61755995 | 50752631 | A | 0.17 | 0.16 | 0.13 | 0.72 | 1.03 | *NIN* |
| 14 | rs2236316 | 50757699 | C | 0.15 | 0.14 | 0.24 | 0.63 | 1.04 | *NIN* |
| 14 | rs2073349 | 50758414 | T | 0.48 | 0.48 | 0.15 | 0.70 | 1.02 | *NIN* |
| 14 | rs77708675 | 50764949 | A | 0.14 | 0.15 | 0.33 | 0.57 | 0.96 | *NIN* |
| 14 | rs72683629 | 50789519 | A | 0.08 | 0.08 | 0.24 | 0.62 | 1.05 | *NIN* |
| 14 | rs17122909 | 50795433 | C | 0.17 | 0.18 | 0.16 | 0.69 | 0.97 | *NIN* |
| 14 | rs12886246 | 50801132 | T | 0.47 | 0.48 | 0.09 | 0.77 | 0.98 | *NIN* |
| 14 | rs34281449 | 50802518 | T | 0.28 | 0.29 | 0.17 | 0.68 | 0.98 | *NIN* |
| 14 | rs72683633 | 50802558 | A | 0.10 | 0.10 | 0.66 | 0.42 | 0.93 | *NIN* |
| 14 | rs113337019 | 50811717 | T | 0.11 | 0.11 | 0.12 | 0.73 | 0.97 | *NIN* |
| **14** | **rs12883458** | **50812661** | **C** | **0.12** | **0.09** | **19.13** | **1.22×10^-5^** | **1.45** | ***NIN*** |
| 14 | rs7153720 | 50813954 | T | 0.27 | 0.28 | 0.15 | 0.70 | 0.98 | *NIN* |
| 14 | rs77367944 | 50815094 | A | 0.25 | 0.26 | 0.56 | 0.46 | 0.96 | *NIN* |
| 14 | rs7149295 | 50816430 | C | 0.37 | 0.36 | 0.17 | 0.68 | 1.02 | *NIN* |
| 14 | rs10132475 | 50819411 | A | 0.15 | 0.15 | 0.27 | 0.61 | 1.04 | *NIN* |
| 14 | rs1951476 | 50822684 | T | 0.13 | 0.13 | 0.46 | 0.50 | 0.95 | *NIN* |
| 14 | rs1547077 | 50823156 | G | 0.16 | 0.16 | 0.10 | 0.76 | 1.02 | *NIN* |
| 14 | rs3759525 | 50826026 | C | 0.10 | 0.09 | 0.42 | 0.51 | 1.06 | *NIN* |
| 14 | rs8018156 | 50826321 | G | 0.11 | 0.10 | 0.79 | 0.37 | 1.08 | *NIN* |
| 14 | rs8010709 | 50828782 | T | 0.40 | 0.39 | 0.33 | 0.57 | 1.03 | *NIN* |
| 19 | rs73050865 | 51612691 | G | 0.06 | 0.05 | 0.55 | 0.46 | 1.09 | *SIGLEC5* |
| 19 | rs73050880 | 51619941 | G | 0.06 | 0.05 | 0.17 | 0.68 | 1.05 | *SIGLEC5* |
| 19 | rs4801882 | 51623800 | G | 0.47 | 0.48 | 0.14 | 0.71 | 0.98 | *SIGLEC5* |
| 19 | rs4802831 | 51623881 | C | 0.06 | 0.07 | 0.67 | 0.41 | 0.91 | *SIGLEC5* |
| 19 | rs8105105 | 51627084 | C | 0.23 | 0.23 | 0.12 | 0.73 | 0.98 | *SIGLEC5* |
| 19 | rs17740650 | 51627235 | G | 0.38 | 0.38 | 0.18 | 0.67 | 1.02 | *SIGLEC5* |
| 19 | rs2278831 | 51627866 | G | 0.14 | 0.15 | 0.33 | 0.57 | 0.96 | *SIGLEC5* |
| **19** | **rs4284742** | **51628480** | **A** | **0.17** | **0.22** | **18.51** | **1.69×10^-5^** | **0.75** | ***SIGLEC5*** |
| 19 | rs556418978 | 51628608 | T | 0.08 | 0.08 | 0.40 | 0.53 | 1.06 | *SIGLEC5* |
| 19 | rs2278833 | 51629807 | G | 0.12 | 0.13 | 0.21 | 0.65 | 0.96 | *SIGLEC5* |

F_A: minor allele frequency in cases; U_A: minor allele frequency in controls. Significant results were highlighted in bold.

Supplemental table S4. Results of haplotype anlaysis.

| Loci | HAPLOTYPE | F_A | F_U | χ^2^ | DF | *P* | SNPS |
| --- | --- | --- | --- | --- | --- | --- | --- |
| *NIN* | OMNIBUS | NA | NA | 0.18 | 2 | 0.91 | rs7494766\|rs61755995 |
| *NIN* | GA | 0.16 | 0.16 | 0.03 | 1 | 0.87 | rs7494766\|rs61755995 |
| *NIN* | GG | 0.31 | 0.31 | 0.18 | 1 | 0.67 | rs7494766\|rs61755995 |
| *NIN* | AG | 0.53 | 0.53 | 0.08 | 1 | 0.78 | rs7494766\|rs61755995 |
| *NIN* | OMNIBUS | NA | NA | 1.25 | 2 | 0.53 | rs2073349\|rs77708675 |
| *NIN* | TA | 0.14 | 0.15 | 0.54 | 1 | 0.46 | rs2073349\|rs77708675 |
| *NIN* | TC | 0.34 | 0.33 | 1.05 | 1 | 0.31 | rs2073349\|rs77708675 |
| *NIN* | GC | 0.52 | 0.52 | 0.20 | 1 | 0.65 | rs2073349\|rs77708675 |
| *NIN* | OMNIBUS | NA | NA | 0.27 | 2 | 0.87 | rs12886246\|rs34281449 |
| *NIN* | TT | 0.28 | 0.29 | 0.25 | 1 | 0.61 | rs12886246\|rs34281449 |
| *NIN* | TG | 0.20 | 0.19 | 0.07 | 1 | 0.79 | rs12886246\|rs34281449 |
| *NIN* | CG | 0.52 | 0.52 | 0.06 | 1 | 0.81 | rs12886246\|rs34281449 |
| *NIN* | OMNIBUS | NA | NA | 0.64 | 2 | 0.73 | rs8018156\|rs8010709 |
| *NIN* | GT | 0.11 | 0.10 | 0.52 | 1 | 0.47 | rs8018156\|rs8010709 |
| *NIN* | AT | 0.29 | 0.29 | 0.04 | 1 | 0.85 | rs8018156\|rs8010709 |
| *NIN* | AG | 0.60 | 0.61 | 0.39 | 1 | 0.53 | rs8018156\|rs8010709 |
| *SIGLEC5* | OMNIBUS | NA | NA | 40.94 | 2 | 1.29×10^-9^ | rs2278831\|rs4284742 |
| *SIGLEC5* | GA | 0.14 | 0.15 | 0.46 | 1 | 0.50 | rs2278831\|rs4284742 |
| *SIGLEC5* | AA | 0.03 | 0.07 | 39.16 | 1 | 3.90×10^-10^ | rs2278831\|rs4284742 |
| *SIGLEC5* | AG | 0.83 | 0.78 | 18.35 | 1 | 1.84×10^-5^ | rs2278831\|rs4284742 |

DF: degree of freedom.

Supplemental Table S5. Significant results in case only association analyses.

| CHR | SNP | BP | A1 | OR | *P* | *OR_adj_ | **P_adj_* |
| --- | --- | --- | --- | --- | --- | --- | --- |
| 19 | rs4284742 | 51628480 | A | 0.31 | 1.06×10^-20^ | 0.30 | 9.60×10^-22^ |
| 19 | rs2278831 | 51627866 | G | 0.33 | 1.12×10^-16^ | 0.32 | 2.25×10^-17^ |
| 19 | rs4801882 | 51623800 | G | 0.54 | 1.43×10^-10^ | 0.54 | 1.14×10^-10^ |
| 19 | rs556418978 | 51628608 | T | 0.40 | 1.25×10^-8^ | 0.39 | 6.10×10^-9^ |
| 19 | rs73050865 | 51612691 | G | 0.44 | 7.05×10^-6^ | 0.43 | 5.81×10^-6^ |
| 19 | rs4802831 | 51623881 | C | 0.50 | 1.31×10^-4^ | 0.48 | 8.88×10^-5^ |
| 19 | rs17740650 | 51627235 | G | 0.73 | 7.18×10^-4^ | 0.73 | 6.76×10^-4^ |

*OR and P values obtained after adjusted by age and gender.

Supplemental table S6. Top 10 significant results for gene by gene interaction analyses.

| CHR1 | SNP1 | CHR2 | SNP2 | STAT | *P* |
| --- | --- | --- | --- | --- | --- |
| 14 | rs10132475 | 19 | rs4284742 | 6.66 | 0.010 |
| 14 | rs113337019 | 19 | rs556418978 | 5.47 | 0.019 |
| 14 | rs10132475 | 19 | rs2278831 | 5.45 | 0.020 |
| 14 | rs72683629 | 19 | rs73050880 | 5.43 | 0.020 |
| 14 | rs7494766 | 19 | rs4801882 | 5.00 | 0.025 |
| 14 | rs3759525 | 19 | rs4801882 | 4.81 | 0.028 |
| 14 | rs77708675 | 19 | rs4801882 | 4.70 | 0.030 |
| 14 | rs3759525 | 19 | rs4284742 | 4.50 | 0.034 |
| 14 | rs3759525 | 19 | rs2278831 | 4.28 | 0.039 |
| 14 | rs1547077 | 19 | rs73050880 | 4.25 | 0.039 |

Supplemental table S7. eQTL data from multiple human tissues for rs12883458 on *NIN*.

| Loci | SNP | *P* | NES | *T*-statistic | Tissue |
| --- | --- | --- | --- | --- | --- |
| *NIN* | rs12883458 | 0.002 | 0.19 | 3.10 | Artery - Tibial |
| *NIN* | rs12883458 | 0.003 | 0.12 | 3.00 | Whole Blood |
| *NIN* | rs12883458 | 0.005 | 0.19 | 2.90 | Muscle - Skeletal |
| *NIN* | rs12883458 | 0.006 | 0.15 | 2.80 | Lung |
| *NIN* | rs12883458 | 0.008 | 0.15 | 2.70 | Cells - Transformed fibroblasts |
| *NIN* | rs12883458 | 0.020 | 0.22 | 2.40 | Colon - Sigmoid |
| *NIN* | rs12883458 | 0.028 | 0.31 | 2.20 | Artery - Coronary |
| *NIN* | rs12883458 | 0.030 | -0.43 | -2.20 | Vagina |
| *NIN* | rs12883458 | 0.034 | 0.23 | 2.10 | Adrenal Gland |
| *NIN* | rs12883458 | 0.037 | 0.15 | 2.10 | Adipose - Subcutaneous |
| *NIN* | rs12883458 | 0.044 | 0.14 | 2.00 | Adipose - Visceral (Omentum) |
| *NIN* | rs12883458 | 0.048 | 0.11 | 2.00 | Esophagus - Muscularis |
| *NIN* | rs12883458 | 0.060 | -0.41 | -1.90 | Brain - Amygdala |
| *NIN* | rs12883458 | 0.064 | 0.18 | 1.90 | Testis |
| *NIN* | rs12883458 | 0.066 | 0.27 | 1.90 | Brain - Frontal Cortex (BA9) |
| *NIN* | rs12883458 | 0.076 | 0.20 | 1.80 | Uterus |
| *NIN* | rs12883458 | 0.120 | -0.19 | -1.60 | Brain - Caudate (basal ganglia) |
| *NIN* | rs12883458 | 0.130 | -0.19 | -1.50 | Brain - Cerebellar Hemisphere |
| *NIN* | rs12883458 | 0.140 | 0.17 | 1.50 | Brain - Cerebellum |
| *NIN* | rs12883458 | 0.150 | -0.21 | -1.40 | Cells - EBV-transformed lymphocytes |
| *NIN* | rs12883458 | 0.160 | 0.11 | 1.40 | Heart - Atrial Appendage |
| *NIN* | rs12883458 | 0.170 | 0.10 | 1.40 | Breast - Mammary Tissue |
| *NIN* | rs12883458 | 0.200 | -0.18 | -1.30 | Brain - Hypothalamus |
| *NIN* | rs12883458 | 0.220 | 0.10 | 1.20 | Esophagus - Mucosa |
| *NIN* | rs12883458 | 0.220 | -0.07 | -1.20 | Skin - Not Sun Exposed (Suprapubic) |
| *NIN* | rs12883458 | 0.230 | 0.10 | 1.20 | Spleen |
| *NIN* | rs12883458 | 0.240 | 0.11 | 1.20 | Colon - Transverse |
| *NIN* | rs12883458 | 0.250 | 0.07 | 1.20 | Thyroid |
| *NIN* | rs12883458 | 0.280 | -0.09 | -1.10 | Artery - Aorta |
| *NIN* | rs12883458 | 0.280 | 0.08 | 1.10 | Heart - Left Ventricle |
| *NIN* | rs12883458 | 0.280 | 0.17 | 1.10 | Liver |
| *NIN* | rs12883458 | 0.290 | -0.06 | -1.10 | Small Intestine - Terminal Ileum |
| *NIN* | rs12883458 | 0.370 | -0.07 | -0.91 | Pituitary |
| *NIN* | rs12883458 | 0.380 | -0.16 | -0.88 | Brain - Putamen (basal ganglia) |
| *NIN* | rs12883458 | 0.430 | -0.14 | -0.80 | Brain - Substantia nigra |
| *NIN* | rs12883458 | 0.450 | 0.09 | 0.76 | Brain - Cortex |
| *NIN* | rs12883458 | 0.460 | -0.13 | -0.74 | Minor Salivary Gland |
| *NIN* | rs12883458 | 0.510 | -0.07 | -0.65 | Prostate |
| *NIN* | rs12883458 | 0.540 | 0.03 | 0.62 | Skin - Sun Exposed (Lower leg) |
| *NIN* | rs12883458 | 0.560 | 0.07 | 0.58 | Brain - Nucleus accumbens (basal ganglia) |
| *NIN* | rs12883458 | 0.560 | 0.07 | 0.58 | Pancreas |
| *NIN* | rs12883458 | 0.720 | 0.07 | 0.36 | Ovary |
| *NIN* | rs12883458 | 0.760 | -0.06 | -0.30 | Brain - Spinal cord (cervical c-1) |
| *NIN* | rs12883458 | 0.780 | 0.02 | 0.28 | Nerve - Tibial |
| *NIN* | rs12883458 | 0.870 | -0.02 | -0.17 | Stomach |
| *NIN* | rs12883458 | 0.920 | 0.02 | 0.10 | Brain - Anterior cingulate cortex (BA24) |
| *NIN* | rs12883458 | 0.920 | 0.02 | 0.10 | Brain - Hippocampus |

NES: normalized effect size.

Supplemental table S8. eQTL data from multiple human tissues for rs4284742 on *SIGLEC5*.

| Loci | SNP | *P* | NES | *T*-statistic | Tissue |
| --- | --- | --- | --- | --- | --- |
| *SIGLEC5* | rs4284742 | 0.004 | -0.098 | -2.900 | Whole Blood |
| *SIGLEC5* | rs4284742 | 0.014 | 0.160 | 2.500 | Nerve - Tibial |
| *SIGLEC5* | rs4284742 | 0.026 | 0.300 | 2.300 | Brain - Putamen (basal ganglia) |
| *SIGLEC5* | rs4284742 | 0.051 | 0.260 | 2.000 | Vagina |
| *SIGLEC5* | rs4284742 | 0.055 | 0.120 | 1.900 | Artery - Aorta |
| *SIGLEC5* | rs4284742 | 0.082 | 0.130 | 1.700 | Heart - Left Ventricle |
| *SIGLEC5* | rs4284742 | 0.087 | 0.190 | 1.700 | Ovary |
| *SIGLEC5* | rs4284742 | 0.120 | 0.170 | 1.500 | Testis |
| *SIGLEC5* | rs4284742 | 0.130 | 0.150 | 1.500 | Adrenal Gland |
| *SIGLEC5* | rs4284742 | 0.180 | 0.120 | 1.400 | Pituitary |
| *SIGLEC5* | rs4284742 | 0.210 | 0.081 | 1.300 | Muscle - Skeletal |
| *SIGLEC5* | rs4284742 | 0.210 | -0.095 | -1.300 | Skin - Not Sun Exposed (Suprapubic) |
| *SIGLEC5* | rs4284742 | 0.220 | 0.180 | 1.200 | Uterus |
| *SIGLEC5* | rs4284742 | 0.250 | 0.095 | 1.200 | Breast - Mammary Tissue |
| *SIGLEC5* | rs4284742 | 0.320 | 0.130 | 1.000 | Brain - Amygdala |
| *SIGLEC5* | rs4284742 | 0.340 | 0.066 | 0.950 | Adipose - Subcutaneous |
| *SIGLEC5* | rs4284742 | 0.350 | 0.047 | 0.930 | Adipose - Visceral (Omentum) |
| *SIGLEC5* | rs4284742 | 0.350 | -0.069 | -0.940 | Esophagus - Muscularis |
| *SIGLEC5* | rs4284742 | 0.350 | 0.076 | 0.940 | Pancreas |
| *SIGLEC5* | rs4284742 | 0.390 | -0.110 | -0.870 | Brain - Frontal Cortex (BA9) |
| *SIGLEC5* | rs4284742 | 0.390 | 0.150 | 0.860 | Brain - Substantia nigra |
| *SIGLEC5* | rs4284742 | 0.410 | 0.110 | 0.820 | Brain - Spinal cord (cervical c-1) |
| *SIGLEC5* | rs4284742 | 0.550 | 0.053 | 0.610 | Colon - Sigmoid |
| *SIGLEC5* | rs4284742 | 0.620 | -0.066 | -0.500 | Brain - Hippocampus |
| *SIGLEC5* | rs4284742 | 0.630 | 0.061 | 0.480 | Brain - Cerebellum |
| *SIGLEC5* | rs4284742 | 0.640 | 0.068 | 0.470 | Brain - Cerebellar Hemisphere |
| *SIGLEC5* | rs4284742 | 0.660 | 0.034 | 0.440 | Spleen |
| *SIGLEC5* | rs4284742 | 0.670 | 0.028 | 0.430 | Colon - Transverse |
| *SIGLEC5* | rs4284742 | 0.680 | 0.028 | 0.410 | Small Intestine - Terminal Ileum |
| *SIGLEC5* | rs4284742 | 0.690 | -0.037 | -0.400 | Artery - Coronary |
| *SIGLEC5* | rs4284742 | 0.720 | -0.046 | -0.350 | Brain - Hypothalamus |
| *SIGLEC5* | rs4284742 | 0.730 | 0.035 | 0.350 | Brain - Anterior cingulate cortex (BA24) |
| *SIGLEC5* | rs4284742 | 0.750 | 0.022 | 0.320 | Heart - Atrial Appendage |
| *SIGLEC5* | rs4284742 | 0.750 | 0.019 | 0.320 | Thyroid |
| *SIGLEC5* | rs4284742 | 0.760 | -0.020 | -0.310 | Artery - Tibial |
| *SIGLEC5* | rs4284742 | 0.780 | -0.031 | -0.280 | Brain - Nucleus accumbens (basal ganglia) |
| *SIGLEC5* | rs4284742 | 0.790 | -0.037 | -0.270 | Cells - EBV-transformed lymphocytes |
| *SIGLEC5* | rs4284742 | 0.830 | 0.011 | 0.220 | Lung |
| *SIGLEC5* | rs4284742 | 0.830 | -0.026 | -0.210 | Minor Salivary Gland |
| *SIGLEC5* | rs4284742 | 0.830 | -0.016 | -0.220 | Stomach |
| *SIGLEC5* | rs4284742 | 0.860 | -0.021 | -0.170 | Brain - Cortex |
| *SIGLEC5* | rs4284742 | 0.870 | 0.010 | 0.160 | Skin - Sun Exposed (Lower leg) |
| *SIGLEC5* | rs4284742 | 0.950 | -0.004 | -0.066 | Esophagus - Mucosa |
| *SIGLEC5* | rs4284742 | 0.990 | 0.001 | 0.010 | Liver |
| *SIGLEC5* | rs4284742 | 0.990 | 0.001 | 0.011 | Prostate |
| *SIGLEC5* | rs4284742 | 1.000 | 0.000 | -0.005 | Brain - Caudate (basal ganglia) |


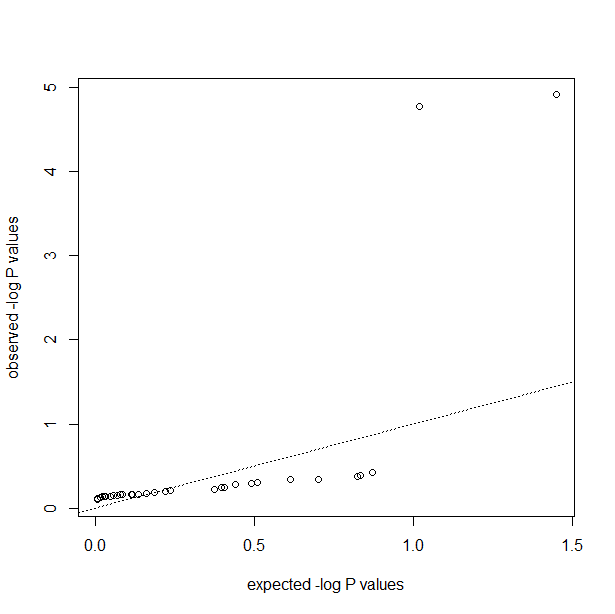


Supplemental figure S1. Q-Q plot for results of single marker based association analyses.


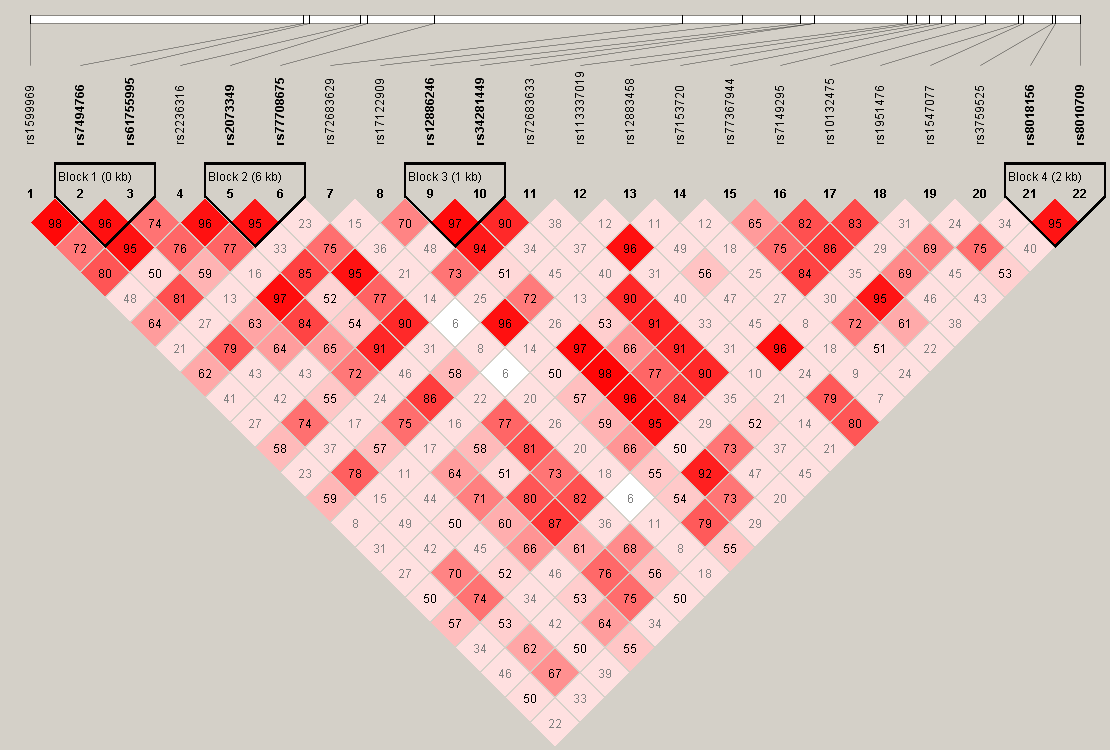


Supplemental figure S2. LD plot for SNPs located on *NIN*. D’ was indicated in each cell.


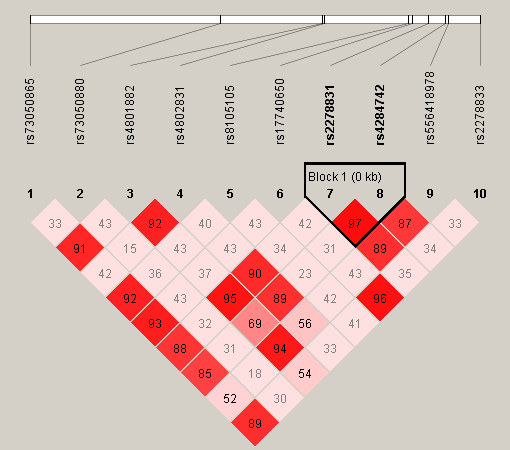


Supplemental figure S3. LD plot for SNPs located on *SIGLEC5*. D’ was indicated in each cell.
